# Buildings Contemplated

**Published:** 1914-08-15

**Authors:** 


### Buildings Contemplated.

Ashburton.—It is proposed to build a new operating theatre and increase the accommodation for private patients at the Ashburton Cottage Hospital.

Beverley.—The East Riding County Council have decided to purchase Ragwell House, near Beverley, at a cost of £6,000, for a tuberculosis sanatorium.

Grays.—The Local Government Board held an inquiry into the application of the Orsett Joint Hospital Board for permission to borrow £4,114, for alterations and additions to their isolation hospital at Little Thurrock. The plans are by Mr. C. M. Shiner, High Street, Grays.

Harwich.—It has been decided to build a Cottage Hospital for Harwich and Dovercourt. The trustees of the late Mr. Whitaker have offered £1,C00 towards the provision of the hospital and £5,000 for endowments, which together with other donations has brought the building fund up to £1,191.

Hollymoor.—The Birmingham Corporation have approved a proposal to extend the Hollymoor Asylum, at an estimated cost of £130,000.

Hyde Hill.—The Metropolitan Asylums Board have purchased fifty-seven acres at Hyde Hill, near Godalming, for building on a portion of the site a tuberculosis sanatorium for women, with accommodation for 200 patients.

Marland.—The Rochdale Corporation have received the sanction of the Local Government Board to a loan of £12,500 for the acquisition of the Springfield Estate at Marland for sanatorium purposes.

Oakdale.—The foundation-stone of the new Cottage Hospital at Oakdale, Sirhowy Valley, has been formally laid.

Plympton St. Mary.—The Local Government Board have approved the plans for the extension of the workhouse infirmary. The amount to be borrowed is £2,975.

Ripon.—The foundation-stone of an extension to the Cottage Hospital has been formally laid. The estimated cost is £1,330. The architect is Mr. H. L. Bown, of Messrs. Bown and Crossland, and the contractor is Mr. A. Trees.

Rugby.—The Guardians have adopted plans for alterations and additions to the workhouse, at an estimated cost of £5,000.

